# Large bronchial artery-pulmonary artery fistula due to cavitating tuberculosis

**DOI:** 10.1093/jscr/rjae405

**Published:** 2024-06-10

**Authors:** Joseph Morrow, Christopher Garvey, Niall McEniff, John Kavanagh

**Affiliations:** Department of Interventional Radiology, St James Hospital Dublin, Dublin 8, Ireland; Department of Interventional Radiology, St James Hospital Dublin, Dublin 8, Ireland; Department of Interventional Radiology, St James Hospital Dublin, Dublin 8, Ireland; Department of Interventional Radiology, St James Hospital Dublin, Dublin 8, Ireland

**Keywords:** tuberculosis, imaging/CT MRI etc, massive haemoptysis, interventional radiology, endovascular embolization

## Abstract

Bronchial artery-pulmonary artery fistulae are rare vascular malformations most commonly caused by infection. Our case presents a 57-year-old male who presented to the Emergency Department with a symptomatic bronchial artery-pulmonary artery fistula due to cavitating pulmonary tuberculosis (TB). The diagnosis was made with multiphase CT angiography of the thorax (including pulmonary and systemic arterial phases). The patient was brought to interventional radiology for further investigation and management. The left upper lobe bronchial artery-pulmonary artery fistula was successfully identified and treated with endovascular embolization. Bronchial artery-pulmonary artery fistulae can pose a diagnostic and therapeutic challenge. Our case demonstrates endovascular embolization as an effective method of treating symptomatic bronchial artery-pulmonary artery fistulae.

## Introduction

Bronchial artery-pulmonary artery fistulae are rare vascular malformations. They most commonly occur due to infection, especially tuberculosis but can be congenital, posttraumatic or caused by malignancy [1–3]. Rasmussen aneurysm, a pulmonary artery pseudoaneurysm created by cavitating tuberculosis is a better known associated condition [4].

We present a case of a patient with a symptomatic bronchial artery-pulmonary artery fistula treated with endovascular embolization.

## Case report

**Figure 1:**
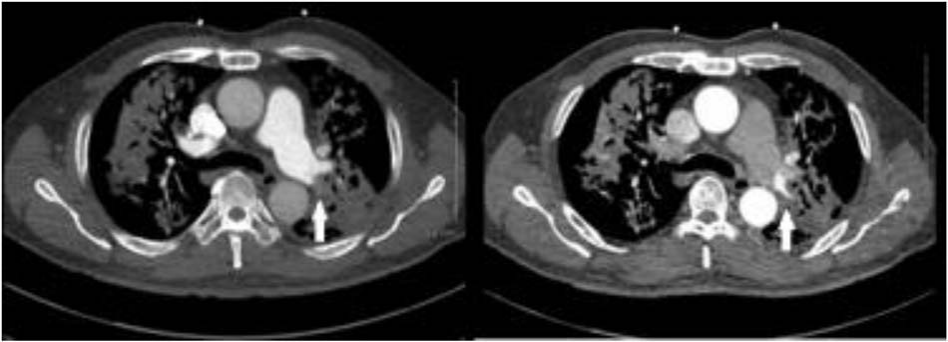
CT pulmonary angiogram (left) demonstrates non-opacifying segmental pulmonary artery (white arrow); on the CT systemic arterial phase angiogram (right), the same segmental pulmonary artery opacifies; background cavitating consolidation is seen in both lungs.

**Figure 2:**
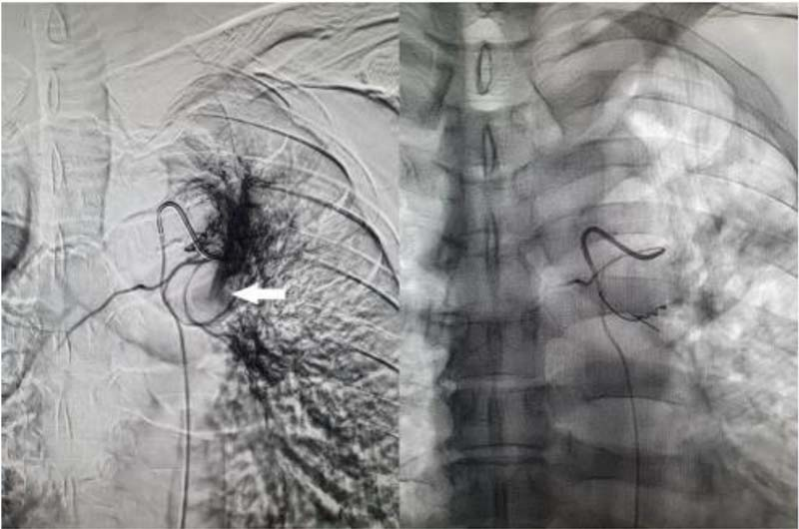
Fluoroscopic image of bronchial artery catheterization and angiogram (left); flow of contrast into the pulmonary artery branch (white arrow) which quickly dissipates, and postembolisation image (right) with microcoils in the treated artery.

A 57-year-old male presented to the Emergency Department unwell with central chest pain, dyspnoea, and haemoptysis. CT pulmonary angiogram demonstrated bilateral cavitating pneumonia. Irregular nonenhancement of a segmental pulmonary artery in the left upper lobe was also noted (Fig. 1). CT thorax in systemic arterial phase demonstrated enhancement of this segmental branch in the left upper lobe (Fig. 1). These findings are due to reversal of flow in the pulmonary artery branch due to higher pressure blood from the bronchial artery passing through the fistula. The patient was brought to interventional radiology for further investigation and management. An initial nonselective descending aorta angiogram demonstrated the bronchial artery-pulmonary artery fistula in the left upper lobe. Selective cannulation of the enlarged left bronchial artery was then performed followed by microcatheter cannulation of the main feeding vessels to the fistula. The vessels were embolized to stasis with 400-μm microparticles (Embozene 400) and microcoils (Fig. 2). The patient tolerated the procedure well and had an uncomplicated postprocedural course. Pansensitive tuberculosis detected on sputum sample. Treatment with Rifater, pyridoxine, and ethambutol were commenced. No further episodes of haemoptysis have been noted to date on clinical follow-up.

## Discussion

Our case demonstrates a symptomatic bronchial artery-pulmonary artery fistula due to cavitating pulmonary tuberculosis. Often these rare entities can pose a diagnostic and therapeutic challenge.

In our case, a pulmonary embolism was initially the primary concern given the presentation of chest pain, dyspnoea, and haemoptysis. It is important to note that in our case, the nonopacification of the pulmonary artery could have been interpreted as a pulmonary embolus if not for the systemic arterial phase imaging. Pulmonary embolus would have fit with the patient’s presentation, but the treatment would be extremely different.

In terms of embolic agent, we opted for 400-μm particles as 355–500 μm particles have proven efficacy and safety profile when used in bronchial arteries [5]. In addition, we opted for spherical shaped particles as they are more uniform in size and penetration characteristic and less prone to clumping in catheters compared with older generation particles. We could have upsized the particles as this was a large shunt; however, the addition of microcoils proved sufficient.

This case demonstrates endovascular embolisation as an effective method of treating symptomatic bronchial artery-pulmonary artery fistulae.
